# Knowledge, attitudes, and practices regarding brucellosis among general population: A cross-sectional study from Jazan Province, Saudi Arabia

**DOI:** 10.5455/javar.2022.i646

**Published:** 2022-12-31

**Authors:** Abdulaziz H. Alhazmi, Asmaa M. Ammar, Fatimah H. Arishi, Abdullah A. Ali, Aisha H. Majrabi, Bahiyyah I. Bahkali, AbdulRahman A. Aqeel, Enas M. Masmali, Yahya J. Alhuraysi, Ahmed M. Albarnawi, Bashair A. Medkhali, Abdulelah A. Mabouj, Ahlam M. Hakami

**Affiliations:** Faculty of Medicine, Jazan University, Jazan, Saudi Arabia

**Keywords:** Brucellosis, zoonosis, Jazan, Saudi Arabia, knowledge, practices

## Abstract

**Objective:**

Brucellosis is an endemic zoonotic infection in Saudi Arabia, including Jazan Province, and epidemiological reports about this disease are limited. In this study, we aimed to investigate knowledge, attitudes, and practices regarding brucellosis among the general population of Jazan Province, southwestern Saudi Arabia.

**Materials and Methods:**

This is a cross-sectional study conducted using a pretested questionnaire that assessed the level of knowledge, attitudes, and practices toward Brucellosis. The calculated sample size was 384, and 1,055 participants were included representing various genders, ages, and levels of education. Data were collected between March and April 2022 and analyzed using descriptive, chi-square, and *t*-test analyses.

**Results:**

Only 50% of the included participants have heard about Brucellosis. Among those who had heard about Brucellosis, 70% had a good knowledge about Brucellosis and that was significantly associated with male gender, being healthcare workers (HCWs), and having a higher level of education. Participants who directly involved in animal care represented 9%, and attitudes and practices regarding brucellosis were varied from average to unsatisfactory. This variation was significantly affected by the level of knowledge.

**Conclusion:**

About 50% of the general population of Jazan Province had never heard about Brucellosis, in a region found prevalent for this disease. Good knowledge was recorded in 70% of those who were aware of Brucellosis, which was significantly associated with the male sex, job as being HCW, and having a higher level of education. These results necessitate public awareness campaign activities to improve knowledge and practices, especially among women, the younger generation, and individuals with limited educational backgrounds. This action could reduce the burden of the disease.

## Introduction

Brucellosis is a highly contagious zoonosis infection caused by bacteria called Brucella [1,2]. Two primary Brucella species that cause human brucellosis and mainly named *Brucella abortus* and *Brucella melitensis* [3,4]. Despite being a reportable disease, reports about the epidemiology of Brucellosis are scarce and this disease is understudied in some parts of the world [2]. It is a zoonotic disease affecting livestock and public health [2,5–7]. Further, Brucellosis is endemic in most developing and limited resources countries, in communities that rely on livestock keeping for a living [6,7].

Clinical manifestation of Brucellosis in animals is often chronic, and can lead to abortion, infertility, and reduced productivity leading to economic loss to the livestock trade [1,8]. In humans, brucellosis is often misdiagnosed with many diseases, or it can be underdiagnosed either due to a lack of medical experts who could diagnose this disease [5,6]. The clinical manifestations of human brucellosis are not specific and can cause muscle and joint pain, fever, malaise, sweating, and chronic hepatomegaly [1,5,6,9]. The complications can occur if left untreated and cases end with arthritis, osteomyelitis, meningitis, and endocarditis and rarely death [2,8]. 

Globally, more than half a million new disease cases are reported yearly, with about 10 per 100,000 population. It remains a significant public health problem in the Mediterranean, western Asia, parts of Africa, and Latin America [10–13]. Seroprevalence rates in countries of the Middle East are varied from 8% to 36% [2,12,13]. In Saudi Arabia, a systematic review was conducted in 2019 and found that the prevalence ranged from 2.3% to 19.2% depending on the region, population, and methods used for the diagnosis [12]. A study was carried out in Jazan Province in 2016 and found that the seroprevalence was 14% [14]. Prevalence was significantly associated with some socioeconomic factors, as it was higher among rural populations, Saudis, and males. Despite this high prevalence rate, studies of knowledge, attitudes, and practices regarding Brucellosis are scarce in the region. This study was conducted to assess awareness, knowledge, attitudes, and, practices regarding brucellosis among the general population in Jazan Region, Saudi Arabia.

## Materials and Methods

### Ethical approval

The ethical consideration to conduct the project was approved by the Jazan University Ethics Committee, with approval number REC-43/07/158, on February 17, 2022. This study was done following the ethical guidelines of the Helsinki Declaration and according to the local recommendation of the National Committee of Bioethics, Saudi Arabia. Participants in this study agreed to participate before data collection. 

### Study design

**Figure 1.**
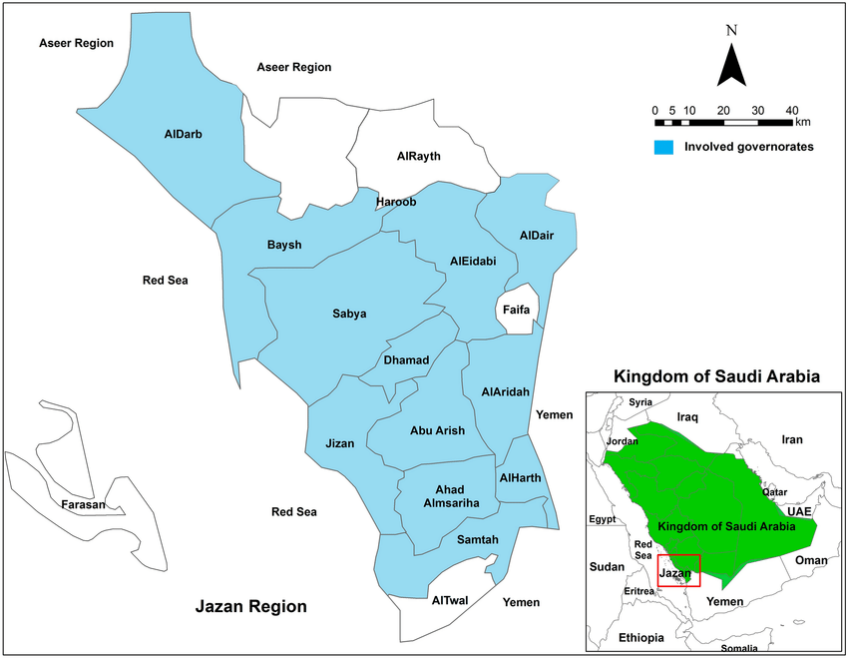
A map of the study area in southwestern region of Saudi Arabia. The map was generated using ArcGIS software version 10.8.1.

A descriptive, cross-sectional study using qualitative data was conducted in Jazan Province, southwestern Saudi Arabia. This region is populated with about 2 million inhabitants, and livestock farming for sheep and other animals is widely practiced mainly (Fig. 1).

### Sampling method, sample size, and inclusion and exclusion criteria

The sample size suggested for this study was calculated to be 384 participants using http://www.raosoft.com/samplesize.html (accessed on February 1, 2022) with a 95% confidence interval, an error rate of less than 5% in a population of two million with a prevalence of 50%. However, we increased the included subjects to 1,055 participants to increase the significant power of this study. Convenient, nonrandom sampling was utilized to reach the study sample. We included adults above 18 years old, of both sexes who lived in Jazan at the time of the study. Those who refused to participate were excluded from this study.

### Data collection tools

A pretested questionnaire [6] was completed using Google Forms as a platform to collect data. The questionnaire contained closed-ended questions that assessed participants’ demographics, knowledge, attitudes, and practices about Brucellosis. Demographic data included age, gender, education level, social status, and occupation. Knowledge of Brucellosis items covers transmission, symptoms, and vaccination. For the knowledge scoring, we used the previously described scoring system [6] in which 11 multiples choice questions were directed to the participant and each correct answer was awarded 1 point. The points were calculated, and the overall score was categorized as (poor) for those who had less than 50% and (good) if the score exceeded 50%. Items connected to attitudes and practices toward Brucellosis covered participants’ general behaviors toward animals and their attitudes toward consuming homemade milk or cheese and their own animals’ meat. A pilot study was done with 20 individuals from the targeted sample to evaluate the questions’ clarity and the duration needed to finish the survey. The results of this pilot study were not included in the final results.

### Data analysis

Data were verified manually, coding was carried out within an Excel sheet, then all data were entered and analyzed using the Statistical Package for the Social Sciences (SPSS version 23). Descriptive statistics were calculated for study variables, e.g., frequency and percentage for qualitative variables and mean and standard deviation for quantitative variables. Tests of significance (e.g., chi-square and *t*-tests) were applied appropriately. *p*-value <0.05 was set to indicate statistical significance.

## Results

### Participants demographics

The included individuals ranged from 18 to 73 years, with a mean age of 28.46 ± 10.2 years. About half of the participants were male adults, 48% were students and 34% were unmarried. Among the adult participants, 72% had university-level education (Table 1). Only 50% have heard about brucellosis and 38% recorded a good knowledge score. 

### Factors affecting the level of awareness

In Table 2, we assessed factors associated with awareness about brucellosis and we found that the mean age of those who have heard about brucellosis was 33.2 + 1.1 compared to those who never heard about it 24.3 + 6.1 (*p*-value = 0.0001). Being male was associated with better awareness about brucellosis compared to females (*p*-value = 0.0001). Regarding jobs, healthcare workers (HCWs) had a better awareness of brucellosis than others (*p*-value = 0.0001). Being married and having two to five children were also linked to awareness about brucellosis compared to other social statuses (*p*-value = 0.0001 for both). Plus, those who have heard about brucellosis usually have a higher educational level and monthly income (*p*-value = 0.0001 for both).

### Level of knowledge and its associated factors

Factors influencing the level of knowledge about brucellosis were evaluated among those who have heard about brucellosis (Table 3). We found that better knowledge was associated with those of mean age of 33.2 years, compared to 25.4 years, and those who had information about Brucellosis from books or other HCWs (*p*-value = 0.0001 and 0.006, respectively).

### Attitudes and practices toward brucellosis

Descriptive data about attitudes and practices toward Brucellosis and their association with the level of knowledge were assessed among those taking care of animals (Table 4). We found that most of the participants usually ate the meat of their animals, and 38% slaughtered their animals by themselves. While 33% of the participants participated in their animals’ birth, 43% never used gloves while dealing with animals. Moreover, we found that the level of knowledge about brucellosis did not significantly affect the attitudes and practices.

## Discussion

One of the common zoonotic diseases is brucellosis, which in endemic regions poses a major threat to public health. Urbanization, the growth of the animal industry, and the lack of hygienic practices in food processing and animal husbandry are reasons why brucellosis is still a threat to the public’s health [5]. Saudi Arabia is one of the countries, where the disease is endemic, with an incidence rate of 7 per 100,000 population, as reported by the Ministry of Health [15]. Yet only a limited number of studies assessed knowledge, attitudes, and practices among the population about brucellosis had been reported in Saudi Arabia, including Jazan [6]. This region is one of the highest population densities, with around 2 million people (Fig. 1). It was previously reported by Ageely et al. [14] that Jazan Province had a high seroprevalence rate (13%) of brucellosis, and this rate was associated with socioeconomic factors. Therefore, this study was conducted to evaluate the level of knowledge, attitudes, and practices about brucellosis.

**Table 1.** Sociodemographic characteristics of the respondents (*n =* 1,055).

Variable		Total	1,055
Age in years: mean | SD		28.86	10.20
		***n***	**%**
Sex	Female	505	48%
	Male	550	52%
Occupation/job-status	Students	511	48%
	HCW	93	9%
	Non-HCW	272	26%
	Looking for Job	103	10%
	Housewife	76	7%
Marital status	Married	395	37%
	Other	660	63%
Residence	Gizan	284	27%
	Abu Arish	146	14%
	Damad	49	5%
	Samtah	71	7%
	Ahad Almasarah	131	12%
	Farasan	40	4%
	Alardah	10	1%
	Fifa	82	8%
	Baish	18	2%
	Addair	6	1%
	Alaidabi	31	3%
	Alhurrath	141	13%
	Sabya	16	2%
	Addarb	6	1%
	Haroub	24	2%
Family members	No children	361	34%
	2–5	309	29%
	5–7	178	17%
	7–10	151	14%
	More than 10	56	5%
Education level	Informal education	8	1%
	High school	239	23%
	University	757	72%
	Postgraduate	51	5%
Family monthly income in SAR	Less than 5k	316	30%
	Between 5k and 10k	293	28%
	More than 10k	446	42%
Heard about Brucella	No	530	50%
	Yes	525	50%
Level of knowledge	Poor	650	62%
	Good	405	38%

SD: Standard deviation. SAR: Saudi Riyals. HCW: Healthcare workers.

In this study, most of the participants were male with a higher level of education, as more than 70% were enrolled in graduate programs. However, half of the participants had not heard about this disease and most of them reported having poor knowledge. In contrast, better knowledge was recorded among older participants and HCWs, mostly due to the nature of their education and training. Another study in the Aseer region, Saudi Arabia, conducted on the older population reported a better level of awareness (73.6%) [6]. Despite its high prevalence in Saudi Arabia [12,14], these findings showed a knowledge gap about brucellosis among study participants and apparently among the younger population in Saudi Arabia, which presents a barrier to its eradication and management as a zoonotic disease. Thus, further efforts are required from the national public health officials in Saudi Arabia to increase awareness about this disease and to ensure the coverage of most cities and villages by these programs to reduce this gap.

Among other socioeconomic factors, educational level affected the awareness and knowledge of Brucellosis. It was observed that individuals with higher educational levels possessed better awareness and good knowledge. This finding was nearly similar to what was reported by others in the region [6,16–18]. For example, in a study conducted in Pakistan where 70% of the individuals had heard about animal brucellosis, and only, 3% were aware of its transmission mode [16]. Furthermore, this knowledge increased with a higher educational level till university (76%), and similar findings were also observed in Yemen and Tajikistan [17,18]. In addition, the level of knowledge was significantly associated with a higher monthly income which might be due to the possession of more access that leads to more information compared to families with less family income. Plus, the source of information seemed to play a role in the level of awareness and knowledge. It was demonstrated that most knowledge was transmitted via social media (31%). However, despite their limited contribution to knowledge transmission (23%), information from HCWs was associated with good knowledge compared to social media (23% vs. 12%). It was obvious that 11% of medical professionals had poor knowledge about Brucellosis, and this finding is in line with others [4,5]. Taken together, Brucellosis should be included in the curricula of medical and public health education, particularly in areas with higher incidence. Further, more information must be delivered to students of health specialties as future healthcare providers to ameliorate public awareness of brucellosis as a mandatory step toward its effective management and elimination [19–21]. 

**Table 2.** Factors affecting awareness about brucellosis.

Variable		Heard about Brucella?				
	Not heard about Brucella (*n* = 530, 50%)		Heard about Brucella (*n* = 525, 50%)		*p*-value	
Age; mean, SD		24	6	33	1	0.0001*
Sex	Female	225	42%	280	53%	0.0001*
	Male	305	42%	245	47%	
Job	Students	338	64%	173	33%	0.0001*
	HCW	26	5%	67	13%	
	Non-HCW	90	17%	182	35%	
	Looking for job	76	14%	103	20%	
Marital status	Married	114	22%	281	54%	0.0001*
	Other	416	78%	244	46%	
Residence	Jazan	145	27%	139	26%	0.0001*
	AbuAriish	83	16%	63	12%	
	Damad	31	6%	18	3%	
	Samtah	39	7%	32	6%	
	Ahad Almasarah	55	10%	76	14%	
	Farasan	36	7%	4	1%	
	Alardah	6	1%	4	1%	
	Fifa	38	7%	44	8%	
	Baish	6	1%	12	2%	
	Addair	0	0%	6	1%	
	Alaidabi	10	2%	21	4%	
	Alhurrath	55	10%	86	16%	
	Sabya	8	2%	8	2%	
	Addarb	3	1%	3	1%	
	Haroub	15	3%	9	2%	
Family members	No children	222	42%	139	26%	0.0001*
	2–5	126	24%	183	35%	
	5–7	85	16%	93	18%	
	7–10	73	14%	78	15%	
	More than 10	24	5%	32	6%	
Education level	Informal education	4	1%	4	1%	0.0001*
	High school	152	29%	87	17%	
	University	360	68%	397	76%	
	Postgraduate	14	3%	37	7%	
Family monthly income in SAR	Less than 5k	203	38%	113	22%	0.0001*
	Between 5k and 10k	150	28%	143	27%	
	More than 10k	177	33%	269	51%	
Level of knowledge	Poor	530	100%	120	23%	0.0001*
	Good	0	0%	405	77%	

SD: Standard deviation. SAR: Saudi riyals. HCW: Healthcare workers.

**Table 3.** Factors affecting the level of knowledge about brucellosis.

Variable Poor		Level of knowledge				*p*-value
		120	Good	405		
Age in years (mean:SD)		25:8		33:12		0.0001*
Sex	Female	58	48%	222	55%	0.214
	Male	62	52%	183	45%	
Job	Students	43	36%	130	32%	0.259
	HCW	13	11%	54	13%	
	Non-HCW	42	35%	140	35%	
	Looking for job	32	27%	81	20%	
Marital status	Married	56	47%	225	56%	0.096
	Other	64	53%	180	44%	
Residence	Jazan	28	23%	111	27%	0.264
	AbuAriish	10	8%	53	13%	
	Damad	7	6%	11	3%	
	Samtah	8	7%	24	6%	
	Ahad Almasarah	21	18%	55	14%	
	Farasan	2	2%	2	0%	
	Alardah	2	2%	2	0%	
	Fifa	10	8%	34	8%	
	Baish	1	1%	11	3%	
	Addair	2	2%	4	1%	
	Alaidabi	3	3%	18	4%	
	Alhurrath	22	18%	64	16%	
	Sabya	1	1%	7	2%	
	Addarb	2	2%	1	0%	
	Haroub	1	1%	8	2%	
Family members	No children	40	33%	99	24%	0.286
	2–5	36	30%	147	36%	
	5–7	18	15%	75	19%	
	7–10	17	14%	61	15%	
	More than 10	9	8%	23	6%	
Education level	Informal education	1	1%	3	1%	0.954
	High school	22	18%	65	16%	
	University	89	74%	308	76%	
	Postgraduate	8	7%	29	7%	
Family income	Less than 5k	27	23%	86	21%	0.354
	Between 5k and 10k	38	32%	105	26%	
	More than 10k	55	46%	214	53%	
Source of knowledge	Friends	57	48%	145	36%	0.006*
	Books	6	5%	42	10%	
	HCW	14	12%	92	23%	
	Social media	43	36%	126	31%	

SD: Standard deviation. SAR: Saudi riyals. HCW: Healthcare workers.

**Table 4.** Attitudes and practices toward Brucellosis.

Variables		Total (*n* = 101) Poor (*n* = 25)		Level of knowledge				*p*-value
				Good (*n* = 76)				
Eating meat from my animals?	Never	13	13%	4	16%	9	12%	0.865
	Sometimes	21	21%	6	24%	15	20%	
	Usually	61	60%	14	56%	47	62%	
	Does not apply	6	6%	1	4%	5	7%	
Slaughter of my animals?	Never	18	18%	5	20%	13	17%	0.720
	Sometimes	37	37%	8	32%	19	25%	
	Usually	28	28%	7	28%	31	41%	
	Does not apply	18	18%	5	20%	13	17%	
Participate in the birth of animals?	Never	31	31%	6	24%	25	33%	0.314
	Sometimes	33	33%	12	48%	21	28%	
	Usually	11	11%	2	8%	9	12%	
	Does not apply	26	26%	5	20%	21	28%	
Use gloves when dealing with animals?	Never	43	43%	9	36%	34	45%	0.081
	Sometimes	21	21%	5	20%	16	21%	
	Usually	8	8%	5	20%	3	4%	
	Does not apply	29	29%	6	24%	23	30%	
Is homemade cheese better than market cheese?	Never	26	26%	5	20%	21	28%	0.849
	Sometimes	20	20%	6	24%	14	18%	
	Usually	22	22%	6	24%	16	21%	
	Does not apply	33	33%	8	32%	25	33%	
Domestic milk is the best?	Never	20	20%	6	24%	23	30%	0.890
	Sometimes	30	30%	7	28%	23	30%	
	Usually	18	18%	5	20%	13	17%	
	Does not apply	24	24%	7	28%	17	22%	

It is crucial to identify high-risk attitudes and practices linked to brucellosis to develop effective control and preventive methods [22]. Similar to a previous study [6], data from this study suggested that most respondents ate meat from their animals, and 38% slaughtered animals by themselves. Further, the consumption of raw milk has already been identified as a risky activity for brucellosis transmission from animals to humans [23]. Our study showed that only 18% believed that domestic milk is the best to be consumed. However, in the study conducted in Pakistan, two third of the respondent reported using raw milk on regular basis, indicating bad hygienic practices [16]. Also, it has been observed in our study, that 28% and 12% of the respondents are sometimes or usually involved in the birth of livestock, and similar findings were observed in a study conducted in Sri Lanka where15.5% of the participants were involved in placenta removal with barehand [1]. Furthermore, the results of this study suggested that only 4% of the respondents used gloves while dealing with animals. Similar data were observed in Tajikistan and Egypt, where participants have reported not using gloves during animal handling [5,24]. These results indicate that the attitudes and practices reported by the participants of this study and as reported by previous studies in the region vary from average to satisfactory, and further enhancement for these practices is crucial through the development and implementation of the right practice programs at regional and national levels [25]. However, to reduce disease transmission, those at risk must be trained at first about handling butchered meat and disposing of ill animals, placentas, and aborted babies. A good attitude toward wearing gloves for protection should be recorded by the appropriate authorities, and livestock caretakers should accept responsibility for utilizing gloves and other measures of protection [12,26].

It is noteworthy that this study was done during the COVID-19 pandemic, a period that directly affected people’s attitude and practice toward zoonotic infections in terms of infection control [27], and very few projects discussed how brucellosis possibly could be a pandemic and the importance of the awareness programs [27]. However, this study was conducted between February and April 2022, when all preventive measures against COVID-19 were reduced in Saudi Arabia [28]. The impact of the COVID-19 pandemic is difficult to be predicted, as a consequence, future studies should be conducted to tackle this area. 

## Conclusion

This study evaluated knowledge, attitudes, and practices of Brucellosis among the general population in Jazan Province, Saudi Arabia, which recorded a high prevalence rate for this zoonotic disease. We found that half of the participants were aware of Brucellosis, and 70% recorded good knowledge. Better knowledge was significantly associated with the male sex, HCW, and those with a higher level of education and income. Awareness programs targeting the general population are required to increase awareness and knowledge, and further national studies about attitudes and practices are essential for a better evaluation of people’s behaviors toward this zoonotic disease. One limitation of this study is that it was conducted in only one province of Saudi Arabia. Thus, more studies are needed at the regional and national levels. Moreover, studies with this type of method have known limitations and biases. Further, questions about practices were not detailed in this study, thus, including more questions on practices related to animal care should be considered in future studies. 

## List of abbreviations

SPSS, Statistical Package for the Social Sciences; HCW, Healthcare workers; SD, Standard deviation; SAR, Saudi riyals.
